# Contribution of membrane raft redox signalling to visfatin-induced inflammasome activation and podocyte injury

**DOI:** 10.18632/aging.205243

**Published:** 2023-11-17

**Authors:** Saisudha Koka, Sreenidhi Surineni, Gurinder Bir Singh, Krishna M. Boini

**Affiliations:** 1 Department of Pharmacological and Pharmaceutical Sciences, College of Pharmacy, University of Houston, Houston, TX 77204, USA; 2 Department of Pharmaceutical Sciences, Irma Lerma Rangel School of Pharmacy, Texas A&M University, Kingsville, TX 78363, USA; 3 Division of Biomedical Sciences, University of California, Riverside, CA 92130, USA

**Keywords:** visfatin, membrane raft, podocyte injury, inflammasome, ROS

## Abstract

Recently we have shown that adipokine visfatin-induced NLRP3 inflammasome activation contributes to podocyte injury. However, the molecular mechanisms of how visfatin-induces the Nlrp3 inflammasome activation and podocyte damage is still unknown. The present study tested whether membrane raft (MR) redox signalling pathway plays a central role in visfatin-induced NLRP3 inflammasomes formation and activation in podocytes. Upon visfatin stimulation an aggregation of NADPH oxidase subunits, gp91phox and p47phox was observed in the membrane raft (MR) clusters, forming a MR redox signalling platform in podocytes. The formation of this signalling platform was blocked by prior treatment with MR disruptor MCD or NADPH oxidase inhibitor DPI. In addition, visfatin stimulation significantly increased the colocalization of Nlrp3 with Asc or Nlrp3 with caspase-1, IL-β production, cell permeability in podocytes compared to control cells. Pretreatment with MCD, DPI, WEHD significantly abolished the visfatin-induced colocalization of NLRP3 with Asc or NLRP3 with caspase-1, IL-1β production and cell permeability in podocytes. Furthermore, Immunofluorescence analysis demonstrated that visfatin treatment significantly decreased the podocin and nephrin expression (podocyte damage) and prior treatments with DPI, WEHD, MCD attenuated this visfatin-induced podocin and nephrin reduction. In conclusion, our results suggest that visfatin stimulates membrane raft clustering in the membrane of podocytes to form redox signaling platforms by aggregation and activation of NADPH oxidase subunits enhancing O_2_^·−^ production and leading to NLRP3 inflammasome activation in podocytes and ultimate podocyte injury.

## INTRODUCTION

The number of obese patients with end stage renal disease has increased significantly worldwide in the last few decades [1]. Obesity results in an increased risk for chronic kidney diseases like diabetes and hypertension which consequently result in chronic kidney disease or even end-stage renal disease [2]. However, the exact mechanism of how obesity increases the advancement of chronic kidney disease is still uncertain. Adipocytes secrete number of bioactive compounds called as “adipokines” [3, 4]. Visfatin is a novel adipokine released from visceral adipose tissue and acts like an inflammatory cytokine. Elevated level of visfatin is reported in inflammation and obesity associated diseases like diabetes, rheumatoid arthritis, inflammatory bowel disease, atherosclerosis, and chronic kidney diseases [5]. Visfatin also induces increased reactive oxygen species generation and proinflammatory cytokines like IL-1β, IL1Ra, Il-6, IL-10 [6]. Recently, altered visfatin levels are reported in chronic kidney diseases and the levels of visfatin increases closely with the severity of endothelial dysfunction, proteinuria, and glomerular dysfunction [7–11]. Also, in population of chronic kidney disease patients, significant elevation of visfatin levels in the plasma is reported [8]. While it has been shown that increased levels of plasma visfatin are associated with the development of CKD and podocyte damage, the precise mechanism by which this adipokine, visfatin, contributes to glomerular sclerosis or end-stage renal disease (ESRD) remains unclear.

In this regard, our group demonstrated recently that visfatin-induced the inflammasome formation and activation in podocytes which subsequently resulted in podocyte damage [12]. However, the exact mechanisms mediating this visfatin-induced inflammasomes formation and activation remains elusive. Numerous mechanisms responsible for the NLRP3 inflammasome activation have been documented, such as lysosome rupture, the activation of ROS (reactive oxygen species) and ion channel gating [13–16]. The NLRP3 inflammasome activation through heightened ROS levels, generally acknowledged and regarded as the most credible mechanism, implies that this inflammasome serves as a universal detector of shifts in cellular oxidative stress. A major source of superoxide in kidney is NADPH oxidase and it consists of five subunits including Rac1/2, gp91phox, p22phox, p47phox, and p67phox [17]. In this study, it is proposed that visfatin induces the NLRP3 inflammasome activation in podocytes, leading to glomerular inflammatory injury in the kidney and the development of CKD, may be primarily driven by NADPH oxidase-mediated membrane raft redox signalling.

## RESULTS

### Visfatin stimulation induces colocalization of p47phox and gp91phox with MR clusters

**Figure 1:**
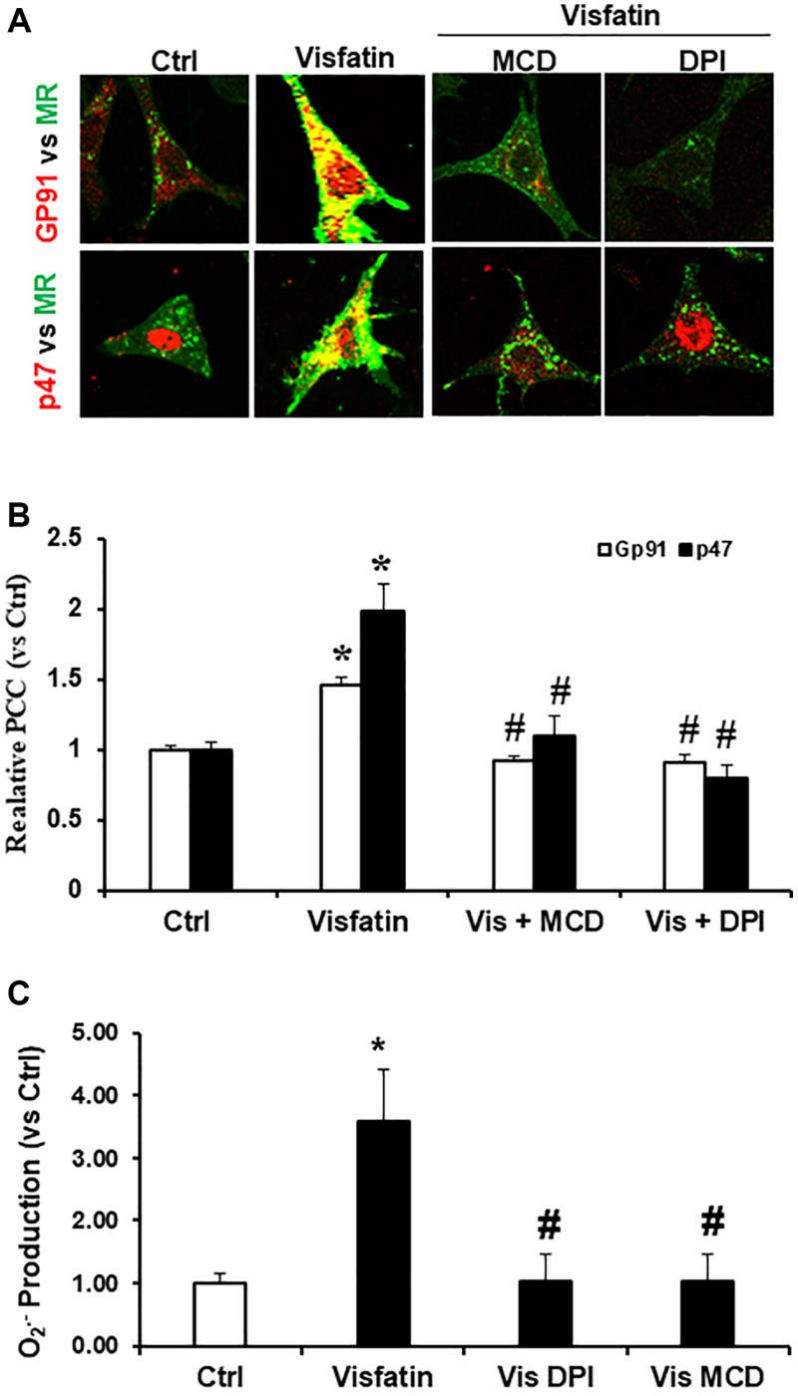
**Effects of visfatin on aggregation of gp91phox, p47phox and MR clusters, O_2_^·−^ production in podocytes.** (**A**) Representative confocal microscopic images of MR clusters (Green) and aggregation of gp91phox (Red) and MR clusters and p47phox (Red) in podocytes (original magnification, 400x). Only overlaid images were presented. Yellow spots in overlaid images indicate co-localization of the MR and gp91phox or p47 phox. (**B**) Summarized data showing the effects of Visfatin, MR disruptor, MCD and NADPH oxidase inhibitors DPI on MR- gp91phox or MR-p47clusters (*n* = 6). Values are means ± SEM, showing fold changes as compared with control. (**C**) Summarized data showing the effects of visfatin on O_2_^·−^ production (*n* = 5 − 7). Values are means ± SEM, showing fold changes as compared with control. Abbreviations: Ctrl: Control; Vis: Visfatin; DPI: diphenyleneiodonium; MCD: methyl-β-cyclodextrin. ^*^P < 0.05 vs. control; ^#^*P* < 0.05 vs. visfatin.

We investigated whether visfatin induces the clustering of membrane rafts (MRs) followed by aggregation and recruitment of NADPH oxidase subunits, specifically gp91phox and p47phox. To assess the relationship of gp91phox and p47phox with MRs, podocyte cells were stained with antibodies against gp91phox or p47phox and Alexa Fluor 488-labeled CTXB for co-localization of MRs and both NADPH oxidase subunits. In Figure 1A, it is evident that CTXB displayed an even distribution on the cell membrane of podocytes treated with the vehicle. However, following visfatin stimulation, MRs appeared as prominent and deep green, fluorescent areas or spots, co-localizing with p47phox and gp91phox. Next, to investigate if these MR clusters play a crucial role in NADPH oxidase subunits recruitment, we examined the impact of the MR disruptor, MCD, on visfatin-induced effects. Our findings revealed that MCD significantly reduced the co-localization of p47phox and gp91phox with MRs (Figure 1A, 1B). Furthermore, we assessed the effect of DPI which is a NADPH oxidase inhibitor to form aggregates of gp91phox within MR clusters. It was observed that DPI markedly reduced the co-localization of gp91phox with MRs, indicating a feedback regulation of MR clustering (Figure 1A, 1B) [18–20].

### Visfatin induces O_2_^·−^ production

Subsequently, we investigated the NADPH oxidase dependent production of O_2_^·−^ in podocytes upon visfatin stimulation, employing the ESR technique. As depicted in Figure 1C, visfatin treatment enhanced O_2_^·−^ generation significantly in podocytes compared to the vehicle treated cells of the control group. However, the visfatin-induced O_2_^·−^ production was notably diminished when podocytes were pre-treated with the MR disruptor MCD or the NADPH oxidase inhibitor DPI, indicating that MR-associated NADPH oxidase subunits were the primary origin of O_2_^·−^ production in response to visfatin treatment.

### Visfatin-induced inflammasome formation and activation were attenuated by inhibiting NADPH oxidase

**Figure 2:**
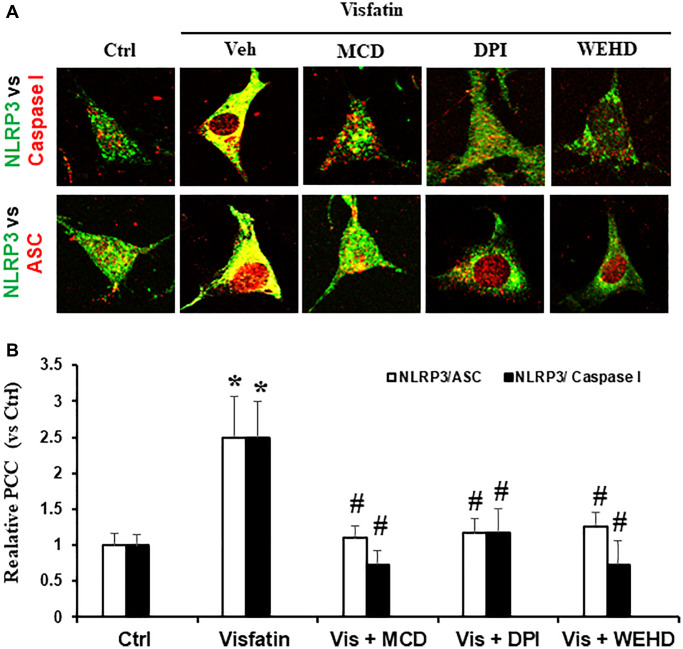
**NADPH oxidase inhibition attenuates inflammasome formation induced by visfatin in podocytes.** (**A**) Confocal images representing the colocalization of NLRP3 (green) with ASC (red) and NLRP3 (green) with caspase-1 (red) in podocytes (original magnification, 400x). (**B**) Summarized data showing the fold change of Pearson correlation coefficient (PCC) for the colocalization of NLRP3 with ASC and NLRP3 with caspase-1 (*n* = 6). Values are means ± SEM, showing fold changes as compared with control. Abbreviations: Ctrl: Control; Veh: Vehicle; Vis: Visfatin; DPI: diphenyleneiodonium; MCD: methyl-β-cyclodextrin. ^*^P < 0.05 vs. control; ^#^*P* < 0.05 vs. visfatin.

**Figure 3:**
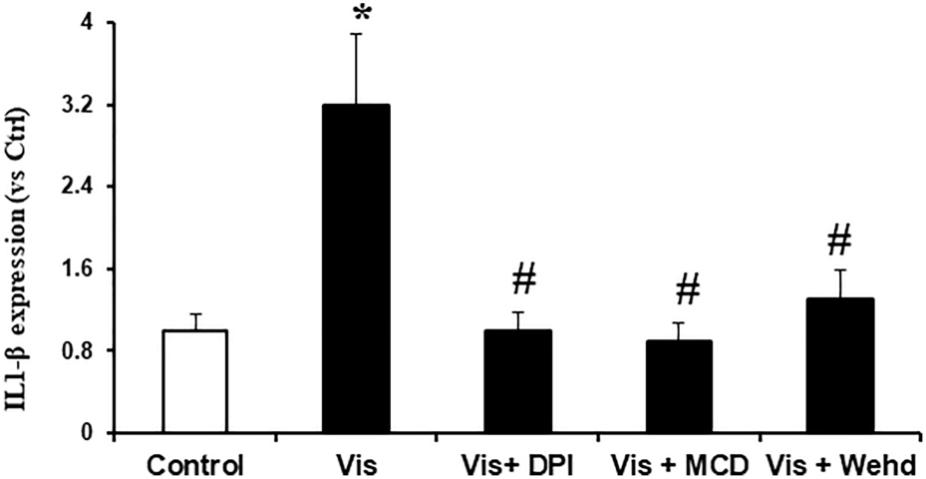
**Effects of NADPH oxidase inhibition on visfatin-induced IL-1β production in podocytes.** IL-1β production in podocytes with or without stimulation of visfatin, DPI or MCD or WEHD. Values are means ± SEM, showing fold changes as compared with control. Abbreviations: Ctrl: Control; Vis: Visfatin; DPI: diphenyleneiodonium; MCD: methyl-β-cyclodextrin. ^*^P < 0.05 vs. control; ^#^*P* < 0.05 vs. visfatin.

We sought to investigate the hypothesis that the activation of NADPH oxidase by visfatin exerts its effects by promoting the NLRP3 inflammasomes formation and activation, consequently contributing to podocyte injury. We investigated whether visfatin had the potential to initiate the assembly and activation of NLRP3 inflammasome complexes in cultured podocytes, by examining the co-localization of NLRP3 inflammasome components and the production of IL-1β. Our confocal microscopy images displayed increased co-localization of inflammasome molecules, as evidenced by the augmented yellow staining (yellow spots) in podocytes when stimulated with visfatin (Figure 2). Pre-treatment of podocytes with the MR disruptor MCD, the NADPH oxidase inhibitor DPI, or WEHD which is the caspase-1 inhibitor effectively hindered the aggregation of NLRP3 with ASC and NLRP3 with caspase-1 induced by visfatin, demonstrating the prevention of inflammasome formation in these cells. NLRP3 inflammasome complex formation leads to the cleavage of pro-caspase-1 protein into its bioactive form, which subsequently binds to and cleaves its substrates, including IL-1β. Corresponding to the confocal microscopy results of inflammasome complex formation, we observed a significant increase in IL-1β production following visfatin treatment (Figure 3). However, this enhanced IL-1β production was mitigated in podocytes pre-treated with the MR disruptor MCD, the NADPH oxidase inhibitor DPI, or the WEHD.

### Inhibition of inflammasomes or NADPH oxidase attenuates the visfatin-induced podocyte injury

**Figure 4:**
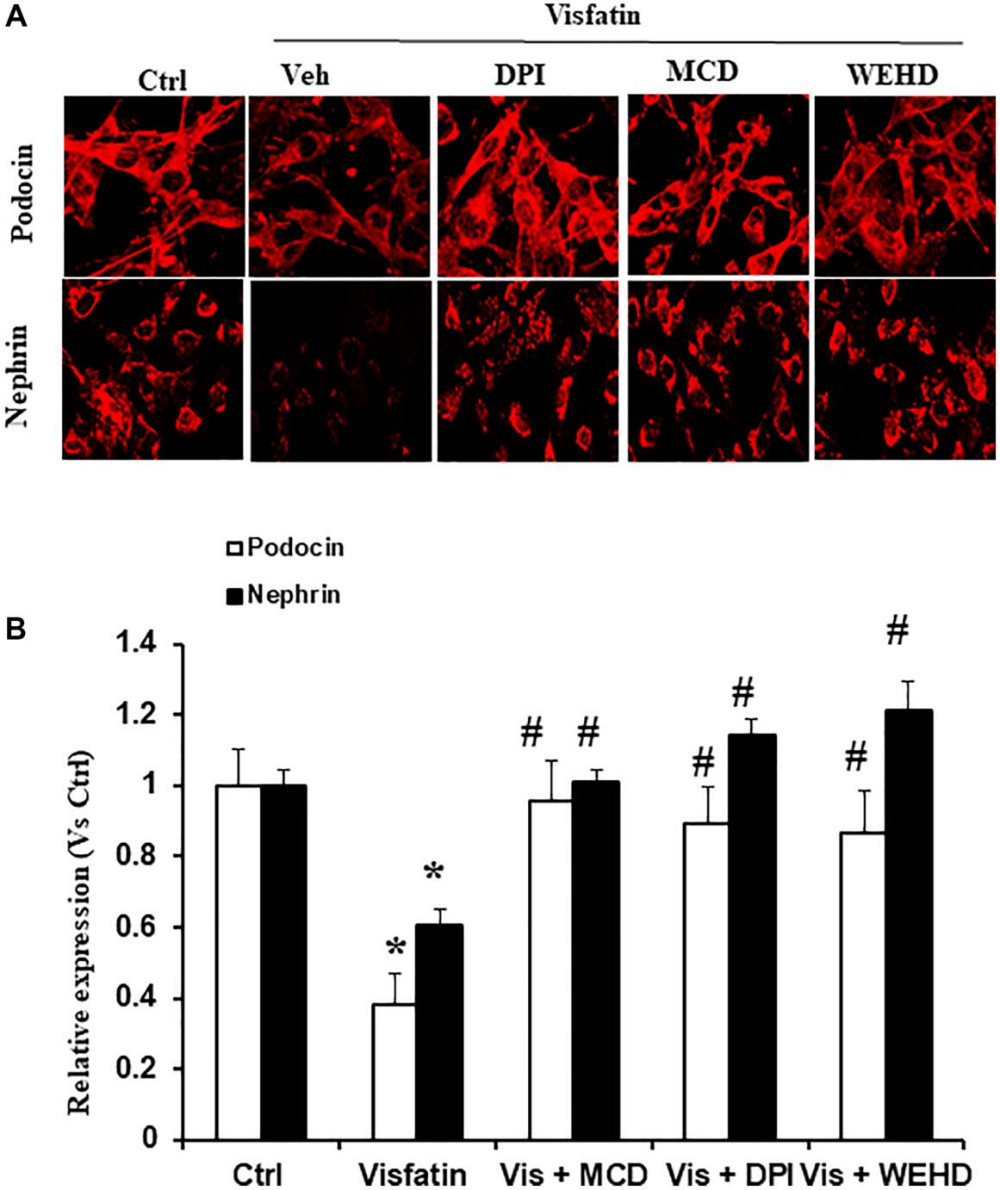
**Effects of Visfatin on podocyte injury.** (**A**) Representative immunofluorescence staining of podocin, nephrin, in podocytes with or without stimulation of visfatin, DPI or MCD or WEHD (original magnification, 400x). (**B**) Summarized data show the percentage of podocyte positive for podocin and nephrin (*n* = 6). Values are means ± SEM, showing fold changes as compared with control. Abbreviations: Ctrl: Control; Veh: Vehicle; Vis: Visfatin; DPI: diphenyleneiodonium; MCD: methyl-β-cyclodextrin. ^*^P < 0.05 vs. control; ^#^*P* < 0.05 vs. visfatin.

Specific markers indicative of podocytes, namely podocin and nephrin, exhibit a decrease in expression levels during podocyte injury [12, 16]. Therefore, we monitored the podocin and nephrin expression to evaluate the extent of podocyte damage. Notably, stimulation with visfatin led to a substantial reduction in the expression of nephrin and podocin, as evidenced by immunofluorescence studies, highlighting significant podocyte damage (Figure 4). Conversely, pre-treatment with the MR disruptor MCD, the caspase-1 inhibitor WEHD, or the NADPH oxidase inhibitor DPI served to protect the podocytes from damage. This was evident through the conserved podocin and nephrin expression levels to a normalized state, comparable to control levels (Figure 4). These outcomes strongly indicate that the redox signaling is mediated by the involvement of NADPH oxidase in the NLRP3 inflammasomes activation and the induction of podocyte dysfunction triggered by visfatin.

### Visfatin induced superoxide production was not inhibited upon inflammasome inhibition

**Figure 5:**
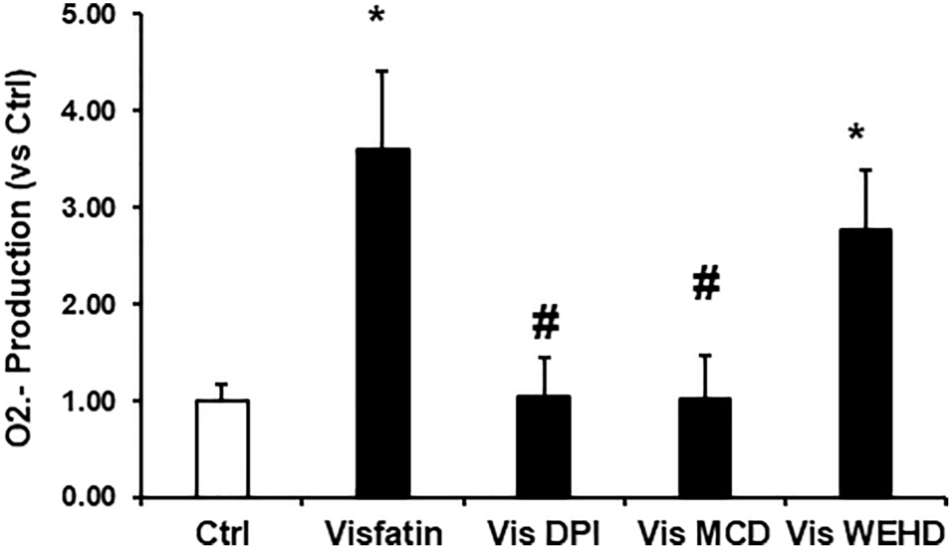
**Effects of NADPH oxidase inhibition on visfatin-induced superoxide production in podocytes.** O_2_^**·−**^ production in podocytes with or without stimulation of visfatin, DPI or MCD or WEHD. Values are means ± SEM, showing fold changes as compared with control. Abbreviations: Ctrl: Control; Vis: Visfatin; DPI: diphenyleneiodonium; MCD: methyl-β-cyclodextrin. ^*^P < 0.05 vs. control; ^#^*P* < 0.05 vs. visfatin.

To further confirm the involvement of NADPH oxidase-derived superoxide in the NLRP3 inflammasomes activation, we assessed superoxide levels in podocytes under different treatment conditions involving visfatin, MR disruptor (MCD), NADPH oxidase inhibitor (DPI), and inflammasome disrupter (caspase-1 inhibitor, WEHD). As depicted in Figure 5, visfatin treatment resulted in a substantial increase in O_2_^·−^ production in podocytes in comparison to control cells. However, this visfatin-induced O_2_^·−^ production was markedly reduced when podocytes were pre-treated with MCD or DPI. Interestingly, the inhibition of inflammasomes had no impact on superoxide production, suggesting that MR-associated NADPH oxidase subunits, which are situated upstream of inflammasomes, served as the primary source of O_2_^·−^ production in response to visfatin stimulation.

### Podocyte monolayer permeability and dysfunction was induced by visfatin

**Figure 6:**
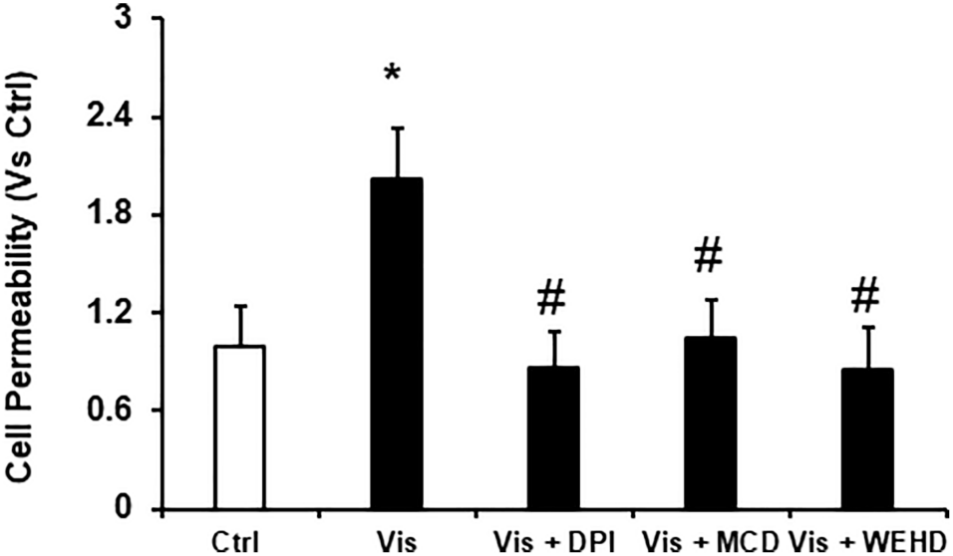
**Inhibition of NADPH and inflammasome attenuates the visfatin-induced cell permeability in podocytes.** Values are arithmetic means ± SEM (*n* = 5 − 8 each group) of cell permeability in podocytes with visfatin in the presence of inhibitors of MR aggregation (MCD), NADPH oxidase inhibitor (DPI) or caspase-1 inhibitor, WEHD. Abbreviations: Ctrl: Control; Vis: Visfatin; DPI: diphenyleneiodonium; MCD: methyl-β-cyclodextrin. ^*^P < 0.05 vs. control; ^#^*P* < 0.05 vs. visfatin.

To delineate the role of visfatin in mediating the dysfunction of podocytes induced by visfatin, we investigated its impact on altering the podocyte monolayers permeability to FITC-dextran. Notably, visfatin treatment led to a significant elevation in podocyte monolayer permeability when assessed against the untreated podocytes. However, this visfatin-induced increase in the permeability of the podocyte monolayer was notably alleviated by prior treatments with the MR disruptor MCD, the NADPH oxidase inhibitor DPI, or the inflammasome disrupter (Caspase-1 inhibitor, WEHD) (Figure 6). These findings provide confirmation that visfatin-associated disruption of the podocyte monolayer operates through the involvement of MR-associated NADPH oxidase subunits.

## DISCUSSION

The fundamental objective of this study was to explore whether the membrane raft (MR) redox signalling pathway plays a role in the NLRP3 inflammasomes activation induced by visfatin, leading to injury of the podocytes. Through experiments conducted on cultured podocytes, we have demonstrated, for the first time that membrane raft-associated redox signalling is essential for the NLRP3 inflammasomes assembly and activation in response to visfatin, subsequently resulting in podocyte dysfunction and injury. These findings shed light on a novel mechanism underlying inflammasome activation and injury of podocytes triggered by visfatin.

NLRP3 inflammasomes serve as an initial mechanism initiating localized inflammatory reactions within the glomeruli. Accumulating evidence suggests that the adipose tissue can also act as an endocrine organ and secretes many bioactive factors, called adipokines [21]. Visfatin, also recognised as NAMPT (extracellular nicotinamide phosphoribosyl transferase) is a 52-kDa novel adipokine that is known to be increased in metabolic disorders and obesity [22]. Visfatin plays a critical role in inflammation and is secreted by activated lymphocytes, neutrophils and monocytes [23, 24]. The function of visfatin in chronic kidney diseases (CKD) is still being investigated and elevated levels of visfatin are shown to be related with CKDs. Many clinical studies have demonstrated the elevation of visfatin levels in patients with CKD [7, 25]. Hsu et al. showed blood concentration of visfatin as an independent predictor of eGFR decline. In both CKD and diabetic studies have shown the increased correlation between visfatin and flow mediated dilation [26]. Previous work by our group showed the role of visfatin in injury of the podocytes [12]. Indeed, the present study demonstrated that visfatin treatment suppressed the expression of nephrin and podocin and increased permeability of podocytes in response to visfatin (Figures 4 and 6). However, it remains unknown how visfatin causes kidney damage and podocyte injury. Recently, it has been demonstrated that visfatin significantly increased superoxide production *in vitro* in glomerular endothelial cells via membrane raft clustering, activating oxidative stress and subsequently alters permeability of the cells leading to kidney disease [18]. Hence, the present study investigated the role of visfatin in activation of membrane raft associated redox signalling platform and subsequent podocyte injury. Hence, first we tested whether visfatin activates the MR-associated redox signalling platform in podocytes. We treated cultured podocytes with visfatin (2 μg/ml) and found that visfatin significantly enhanced the aggregation of MR in podocytes in response to visfatin as compared to untreated podocytes (data not shown). Further we found that visfatin increased colocalization of MR with gp91phox and MR with p47phox in podocytes as compared to untreated control podocytes. Visfatin associated MR aggregation and subsequent NADPH oxidase activation were abolished upon treatment of podocytes with MR disrupter MCD and NADPH oxidase inhibitor DPI. These findings indicate that, in response to visfatin stimulation in cultured podocytes (Figure 1A, 1B), NADPH oxidase subunits such as gp91phox and p47phox appear to aggregate. To further substantiate the link between membrane raft (MR) associated aggregation of NADPH oxidase components and enzyme activation, using ESR technique we assessed NADPH oxidase-derived O_2_^·−^ production in podocytes. We observed a significant increase in O_2_^·−^ production in podocytes upon treatment with visfatin, and this elevated O_2_^·−^ production was mitigated when podocytes were treated with the MR disrupter MCD and the NADPH oxidase inhibitor DPI (Figure 1C). These results collectively provide the first evidence that visfatin induces MR clustering, leading to the subsequent assembly and aggregation of NADPH oxidase subunits, resulting in the formation of an active enzyme complex that produces O_2_^·−^ and contributes to podocyte injury.

Next, we examined the possible mechanism of how visfatin-induced MR aggregation and subsequent NADPH oxidase activation leads to podocyte injury. Recently, Koka et al. reported that visfatin can activate NLRP3 inflammasomes in podocytes and contributes to podocyte injury [12]. The NLRP3 inflammasome functions as a detector and has been demonstrated to participate in both inflammatory and non-inflammatory reactions [27, 28]. In many inflammatory diseases like diabetes, obesity, atherosclerosis and CKD, the role of inflammasome activation has been reported [29–33]. The mechanisms of activating NLRP3 inflammasomes in response to visfatin are still not explored. Therefore, we sought to examine whether the activation of MR-associated NADPH oxidase by visfatin contributes to the initiation of inflammasome activation. In this context, prior studies have firmly established that the reactive oxygen species (ROS) is involved in the NLRP3 inflammasomes activation. The activation of the NLRP3 inflammasome in response to elevated ROS levels is widely acknowledged as the most accepted and plausible mechanism [34, 35]. Remarkably, our findings demonstrate an increase in IL-1β production and the co-localization of NLRP3 with caspase-1 or NLRP3 with ASC upon visfatin stimulation, indicating the formation and activation of NLRP3 inflammasomes in podocytes. Significantly, this visfatin-induced inflammasome assembly and activation were nullified in podocytes pre-treated with MCD, DPI, or WEHD (Figures 2 and 3). These results unequivocally indicate that the assembly of NLRP3 inflammasomes and activation in podocytes, facilitated by MR-associated NADPH oxidase, may cause the development of kidney inflammatory diseases in response to visfatin.

Furthermore, we investigated the involvement of NADPH oxidase-derived O_2_^·−^ in visfatin-triggered NLRP3 inflammasome activation. Our observations revealed that visfatin treatment substantially increased O_2_^·−^ production in podocytes compared to the control cells. Notably, the visfatin-induced O_2_^·−^ production was significantly reduced when podocytes were pre-treated with MCD or DPI. Interestingly, the inhibition of inflammasomes (using WEHD, a caspase-1 inhibitor) had no impact on superoxide production, indicating that MR-associated NADPH oxidase subunits, which are upstream of inflammasomes, served as the primary source of O_2_^·−^ production in response to visfatin stimulation. Further, we explored the functional importance of visfatin-induced membrane raft-associated redox signaling in NLRP3 activation by examining the effect of visfatin on the permeability of the podocyte monolayer. Enhanced vascular permeability, leading to increased glomerular permeability, is recognized as a contributing factor in glomerular injury [18, 36]. Our study has revealed that visfatin elevated the podocyte cell permeability, and this increase was significantly mitigated by treatment with the MR inhibitor MCD, the NADPH oxidase inhibitor DPI, and the caspase-1 inhibitor WEHD. This suggests the involvement of MR-NADPH-NLRP3 signaling in the visfatin-induced rise in podocyte permeability (Figure 6).

In conclusion, our findings suggest that visfatin induces the clustering of membrane rafts in podocyte membranes, forming redox signaling platforms through the activation and aggregation of NADPH oxidase subunits. This process enhances O_2_^·−^ production and ultimately leads to NLRP3 inflammasome activation in podocytes, resulting in podocyte injury.

## MATERIALS AND METHODS

### Cell culture

Podocytes were cultured and maintained as we described previously [12]. Podocytes were subjected to treatment with visfatin (2 μg/ml), either alone or in combination with inhibitors including NADPH oxidase inhibitors like diphenylene iodonium (DPI, 10 μM), membrane raft disruptors such as methyl-β-cyclodextrin (MCD, 1 mM), and the caspase I inhibitor Z-WEHD-FMK (WEHD, 1 mmol/L). The concentration and incubation duration of visfatin in cell culture dishes were selected based on previous studies [18].

### Confocal microscopy

To visualize membrane rafts (MR) and their associated proteins for inflammasome formation in podocytes via confocal microscopy, the following procedure was followed: Podocytes were cultured on poly-L-lysine-coated chambers and treated with visfatin, DPI, MCD, or WEHD as described, overnight. The cells were then rinsed with cold PBS, fixed in 4% paraformaldehyde for 15 minutes, and subsequently blocked with 1% BSA in PBS for 30 minutes. To stain MRs, Alexa488-labeled cholera toxin B (CTXB; 1 μg/mL, Thermo Fisher Scientific, USA, Cat # C22841) was applied for one hour. For the assessment of MR colocalization with gp91phox and p47phox, podocytes were incubated overnight with primary antibodies, including anti-gp91phox (1:200 dilution, Santa Cruz, USA, Cat # Sc35503) and anti-p47phox (1:200 dilution, Santa Cruz, USA, Cat # Sc376614) for mouse antibodies. To detect the colocalization of inflammasome molecules, podocytes were incubated with rabbit anti-NLRP3 (1:200 dilution, Abcam, USA, Cat # Ab4207) and mouse anti-ASC (1:200 dilution, Santa Cruz, USA, Cat # Sc514414), or rabbit anti-NLRP3 (1:200, Abcam, USA, Cat # Ab4207) and anti-caspase-1 (1:200, Santa Cruz, USA, Cat # Sc56036). To assess podocyte injury, podocytes were incubated overnight with rabbit anti-podocin (1:200 dilution, Sigma-Aldrich, USA, Cat # PO372) or mouse anti-nephrin (1:200 dilution, Santa Cruz, USA, Cat # Sc377246) antibodies. After washing, the slides containing probed primary antibodies were treated with Alexa-488- or Alexa-555-labeled secondary antibodies for 1 hour at room temperature (Invitrogen, Carlsbad, CA, USA). Subsequently, the slides were mounted with a DAPI-containing mounting solution and examined using a confocal microscope (Leica, Buffalo Grove, IL, USA). Images were captured and analyzed the colocalization of MR with p47phox or MR with gp91phox or NLRP3 with ASC or NLRP3 with caspase-1 using Image Pro Plus 6.0 software (Media Cybernetics, Bethesda, MD, USA) [15].

### Cell permeability assay

Podocytes were cultured in 24-well transwell plates. After the designated treatments, the transwell inserts were transferred to unused wells containing 200 μL of fresh medium. Each insert received 100 μL of FITC–dextran (10 kDa, Sigma-Aldrich, St. Louis, MO, USA) and was then incubated at 37°C for 2 hours. Subsequently, the inserts were extracted and the fluorescent intensity was measured at wavelength of 485/530 nm (excitation/emission) using a fluorescent microplate reader (Biotek Instruments, Winooski, VT, USA). The arbitrary fluorescence intensity was employed to compute the relative permeability [37].

### IL-1β production

The quantification of IL-1β production in podocyte supernatant was conducted using a commercially accessible ELISA (R&D Systems, Minneapolis, MN, USA) [33].

### Electronic spin resonance (ESR) analysis of O_2_^·−^ production

The electronic spin resonance detection of O_2_^·−^ (superoxide) was carried out as we previously outlined [12, 18]. In brief, to assess NADPH dependent O_2_^·−^ production, proteins extracted from podocytes were resuspended in a modified Kreb’s-Hepes buffer containing deferoxamine (100 mM, Sigma-Aldrich) and diethyldithiocarbamate (5 mM, Sigma-Aldrich). The Nox-dependent O_2_^·−^ production was evaluated by introducing 1 mM NADPH as a substrate to 50 mg of protein, followed by incubation for 15 minutes at 37°C, either with or without the presence of SOD (200 U/ml). Subsequently, 1 mM of the O_2_^·−^ specific spin trap, 1-hydroxy-3-methoxycarbonyl-2,2,5,5-tetramethylpyrrolidine (CMH, Noxygen, Elzach, Germany), was added. The resulting mixture was loaded into glass capillaries and immediately subjected to kinetic analysis of O_2_^·−^ production over a 10-minute period using a Miniscope MS200 electromagnetic spin resonance (ESR) spectrometer (Magnettech Ltd., Berlin, Germany). The findings were expressed as fold changes relative to the control [12].

### Statistical analysis

The data is presented as the arithmetic mean ± SEM, with ‘n’ denoting the number of distinct experiments. Significance testing was conducted using ANOVA or, where relevant, paired, and unpaired Student’s *t*-tests. Statistically significant results were defined as those with a *p*-value less than 0.05.
